# Recent Progress in Cellulose-Based Conductive Hydrogels

**DOI:** 10.3390/polym17081089

**Published:** 2025-04-17

**Authors:** Zhenrui Du, Na Wang, Jie Du

**Affiliations:** School of Materials Science and Engineering, Hainan University, Haikou 570228, China; 20223000742@hainanu.edu.cn (Z.D.); 20223000826@hainanu.edu.cn (N.W.)

**Keywords:** cellulose, conductive hydrogels, biomaterials, flexible electronics, multifunctionality

## Abstract

Cellulose, a widely abundant natural polymer, is well recognized for its remarkable properties, such as biocompatibility, degradability, and mechanical strength. Conductive hydrogels, with their unique ability to conduct electricity, have attracted significant attention in various fields. The combination of cellulose and conductive hydrogels has led to the emergence of cellulose-based conductive hydrogels, which show great potential in flexible electronics, biomedicine, and energy storage. This review article comprehensively presents the latest progress in cellulose-based conductive hydrogels. Firstly, it provides an in-depth overview of cellulose, covering aspects like its structure, diverse sources, and classification. This emphasizes cellulose’s role as a renewable and versatile material. The development and applications of different forms of cellulose, including delignified wood, bacterial cellulose, nanocellulose, and modified cellulose, are elaborated. Subsequently, cellulose-based hydrogels are introduced, with a focus on their network structures, such as single-network, interpenetrating network, and semi-interpenetrating network. The construction of cellulose-based conductive hydrogels is then discussed in detail. This includes their conductive forms, which are classified into electronic and ionic conductive hydrogels, and key performance requirements, such as cost-effectiveness, mechanical property regulation, sensitive response to environmental stimuli, self-healing ability, stable conductivity, and multifunctionality. The applications of cellulose-based conductive hydrogels in multiple areas are also presented. In wearable sensors, they can effectively monitor human physiological signals in real time. In intelligent biomedicine, they contribute to wound healing, tissue engineering, and nerve regeneration. In flexible supercapacitors, they offer potential for green and sustainable energy storage. In gel electrolytes for conventional batteries, they help address critical issues like lithium dendrite growth. Despite the significant progress, there are still challenges to overcome. These include enhancing the multifunctionality and intelligence of cellulose-based conductive hydrogels, strengthening their connection with artificial intelligence, and achieving simple, green, and intelligent large-scale industrial production. Future research directions should center around exploring new synthesis methods, optimizing material properties, and expanding applications in emerging fields, aiming to promote the widespread commercialization of these materials.

## 1. Introduction

The advent of a plethora of multifunctional, innovative materials has led to the proliferation of flexible electronics across a multitude of emerging industries [1]. These include, but are not limited to, electronic skin, wearable flexible sensors, soft robots, intelligent Internet of Medical Things (IoMT) applications, and more [2]. The confluence of flexible electronics with the advancement of sensing technology and artificial intelligence promises to enhance human well-being in a multitude of domains [3]. The global flexible electronics market, valued at USD 28.9 billion in 2022, is projected to grow exponentially to USD 144.3 billion by 2030 (CAGR: 22.3%), driven by applications in healthcare, consumer electronics, and environmental monitoring. Cellulose-based conductive hydrogels are poised to address critical challenges in these sectors, including biocompatibility, sustainability, and cost efficiency, with potential contributions to a USD 195 billion medical wearable market and USD 40 billion IoT sensor industry by 2030. The original traditional rigid materials through the structure and process improvement make rigid electronic devices with deformability. However, the enhanced rigid material will nevertheless encounter a mechanical mismatch when coupled with a malleable substrate, such as human skin, which will consequently result in adverse effects, including signal fluctuation, unstable performance, and human body unfitness when the device is utilized in practice [4]. In this context, flexible electronic materials will be lighter, more compact, and have a human skin conformal connection. This will allow them to overcome the shortcomings of traditional rigid electronic devices, better meet the needs of the Internet of Everything and human–machine combination, and better meet the needs of people in their daily lives for a deeper level of artificial intelligence applications [5]. To illustrate, flexible sensors are capable of accepting a multitude of physical, chemical, environmental, and other stimuli. When combined with machine learning technology and an integrated artificial intelligence system that simulates the function of the human nervous system, these sensors have the potential to revolutionize numerous fields, including medical care, robotics, sports, and the smart home [6,7].

Conductive hydrogel is very good for overcoming the incompatibility of electronics and biology. Its characteristics include a three-dimensional mesh structure, hydrophilic water retention, and biocompatibility, with excellent conductivity to achieve wireless transmission of bioelectric signals [8]. This material has been employed in a range of applications, including drug delivery, wound dressings, tissue regeneration, and health monitoring [9]. Concurrently, cellulose, a highly prevalent natural polymer, offers a promising alternative to petroleum-based materials [10]. Its exceptional mechanical strength, electrical conductivity, chemical reactivity, biocompatibility, and degradability make it an excellent candidate for the development of conductive hydrogels. Cellulose and its derivatives have been employed in a multitude of fields, including the biomedical field, food, electronic components, new energy, and numerous others [11]. The development of cellulose-based conductive hydrogels is also attracting considerable research interest as a means of creating flexible conductive materials with a range of potential applications in fields such as medicine, agriculture, and the environment. The objective of this paper is to examine the actual development and application status of cellulose-based conductive hydrogels.

## 2. Development and Use of Cellulose

### 2.1. Overview of Cellulose

Cellulose is one of the most common natural polymers in nature. It is a renewable resource derived from a wide range of sources, with an annual output that is both high and low cost. Additionally, cellulose exhibits natural degradation and other characteristics that make it an excellent choice to replace petroleum-based materials. Taking electronic products as an example, the recycling and treatment of electronic waste has always been a major problem that needs to be solved urgently in our country. The environmental pollution generated under the huge demand has always been a problem that needs to be solved in the face of environmental pollution in our country [12]. It is therefore of great significance to develop and utilize natural polymer functional materials, exemplified by cellulose. Cellulose is derived from a multitude of natural resources, including wood, bamboo, cotton, algae, and bacteria [13]. In the current situation of global development, reducing the production of cellulose from wood as a raw material not only better meets the requirements of carbon neutrality and environmental protection, but also has great application prospects. For example, cereals such as maize and wheat are widely grown around the world, and straws, which are agricultural residues, are a potential choice for cellulose production [14]. Liang J. et al. fully utilized maize stover by pre-hydrolysis and alkaline sulfite cooking [15]. Fitria et al. found that the chemical composition of stover from 30 wheat varieties ranged from 33.7% to 36.3% glucan (for cellulose), 16.8% to 19.5% xylan (for hemicellulose), and 18.4% to 20.6% lignin, respectively. Furthermore, the differences in stover yield and potential sugar yield per unit area were explored. This research is informative with respect to the selection of raw materials for cellulose production [16]. Concurrently, the ethanol-based organosolv fractionation of wheat straw by Wildschut J et al. is also of significant value for the cellulose production process [17]. Water hyacinth (*Eichhornia crassipes*), which is one of the main items to be treated for river pollution, can also be used for cellulose production. Afdhal et al. employed a synergistic combination of steam explosion pretreatment and optimized ultrasonic fibrillation to produce cellulose nanofibers (CNFs) from Eichhornia crassipes, while Hemida et al. produced crystalline cellulose nanocrystals (CNCs) by acid hydrolysis [18,19]. Some organic matter in municipal solid waste, such as waste paper, can also be used for cellulose production. Waste paper isolated from mixed municipal solid waste (MSW) has been identified as a potential starting material for the production of CNFs by Hietala et al. [20]. There is still a large number of residues or wastes that can replace wood in the production of cellulose, waiting to be explored and exploited by scientists. Additionally, a substantial number of natural cellulose molecules with hydroxyl groups can undergo esterification and etherification through chemical reactions, resulting in the generation of cellulose ethers and cellulose esters, among other derivatives. As the representative of a series of cellulose derivatives, it is capable of meeting the demand for research and the development of a variety of products and materials. It has been industrialized and applied in hundreds of species, and further reduction in the molecular size of cellulose allows the formation of bacterial cellulose nanocellulose (BNC), fibrous CNCs, long and flexible CNF, and a series of derivatives. Consequently, the development of cellulose-based functional materials through a variety of processing means, based on the ease of processing, biocompatibility, mechanical strength, degradability, and chemical properties of cellulose, will result in a multitude of potential applications and significant scientific value. This section will examine the potential and value of cellulose from the perspectives of its structure, source, and classification.

### 2.2. Structure of Cellulose

**Figure 1:**
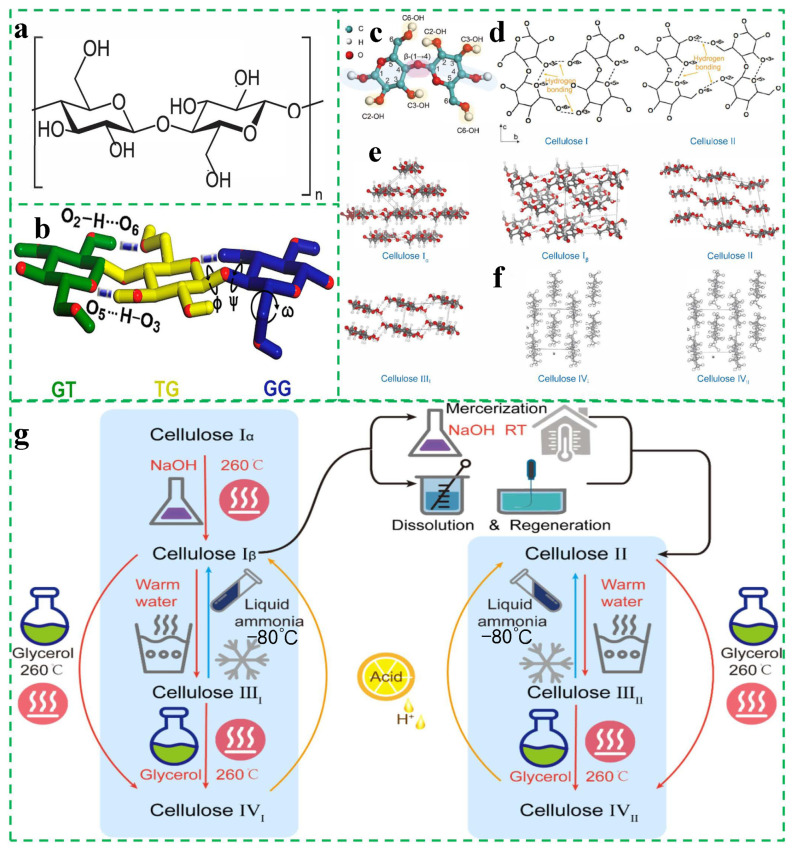
Structure of cellulose. (**a**) Molecular structure of cellulose. (**b**) Stereo model of three selected D-glucose monomers in the cellulose backbone. The model omits the aliphatic hydrogen atoms and shows the oxygen atoms in red. The rotation of the glycosidic bonds is shown and is used as a measure of the order of the cellulose chain [21]. Copyright 2019, ELSEVIER. (**c**) Molecular model of cellulose. (**d**) Hydrogen bonding patterns of cellulose I and cellulose II. (**e**) Crystal structure models of cellulose I, II, and III. (**f**) Projection in the [0 0 1] direction of the parallel chains of the cellulose IVI plane (left) and the antiparallel corner and central chains of cellulose IV_II_ (right) on the a–b base plane. (**g**) Interconversion between different crystal structures of cellulose [24]. Copyright 2023, ELSEVIER.

Cellulose is a linear polysaccharide polymer, a long-chain macromolecule formed from glucose units linked by β-1,4 glycosidic bonds. As illustrated in Figure 1a,b, the fundamental unit of the cellulose molecular chain, D-anhydroglucopyranose, is in the most stable chair conformation and comprises two β-D glucose bases that are planarly linked to each other in a 180° manner [21,22].

Cellulose molecules are characterized by the presence of reactive groups, with three reactive hydroxyl groups per basic unit. This confers upon them a distinctive chemical reactivity, coupled with a high capacity for hydrogen bonding interactions. The formation of hydrogen bonding networks by cellulose molecular chains gives rise to a variety of distinctive physical and chemical properties. To illustrate, hydrogen bonding causes the cellulose molecular chain to be closely arranged. At room temperature, cellulose is soluble neither in water nor in general organic solvents, including ethanol, acetone, benzene, and so forth. The stable intramolecular and intermolecular hydrogen bonding network endows cellulose with a high degree of chemical stability. Hydroxyl modification can be activated by a variety of methods, including physical, chemical, and biological approaches. Wang et al. has proposed a strategy for reconstructing the hydrogen bonding network into a dynamic covalent adaptive network, which effectively mitigates the influence of the hydrogen bonding network and facilitates the development of cellulose bioplastics through thermal processing [23].

The aggregated state of cellulose molecules is characterized by the ease of their crystallization and the formation of a prefibrillar structure, which can be attributed to the action of hydrogen bonding and van der Waals forces on linear cellulose molecular chains. Cellulose is composed of two distinct phases: crystalline and amorphous. This is illustrated in Figure 1d–f. The crystalline structure of cellulose comprises five distinct structures: cellulose I (natural cellulose), cellulose II, cellulose III, cellulose IV, and cellulose X (an artificial class of cellulose). Of these, cellulose II, III, IV, and X have been synthesized and can be transformed from cellulose I under certain conditions. The parallel stacking of multiple cellulose molecular chains gives rise to the formation of basic protofibers, which can subsequently undergo aggregation to form microcellulose fibers that exhibit high axial strength [24,25,26].

### 2.3. Sources and Classification of Cellulose in Practical Applications

Cellulose is derived from renewable natural resources, including wood, cotton, algae, and bacteria. Nevertheless, the structure and dimensions of natural cellulose exhibit some degree of variation contingent on the source. Cellulose exhibits a hierarchical structure, with variations in mechanical strength and functionality observed across different size ranges. In the case of gel-like materials, the selection of cellulose raw materials and the construction of the size are of great importance in order to meet different application scenarios and functional requirements. In this section, we present a sequential discussion of the sources and applications of macroscopic-sized delignified wood, micro- and nano-sized bacterial cellulose, nano-sized nanocellulose, and molecular-sized modified cellulose in gels.

Delignified wood (DW) is produced by the removal of lignin from wood through the use of acids, alkalis, and enzymes. DW exhibits ultra-high mechanical strength in the direction of fiber growth, lower thermal conductivity, and a greater number of free hydroxyl groups on cellulose than natural wood. The cellulose networks found in DW are ideal for use in biomass-reinforced components, as demonstrated by Wang et al. A biomimetic strategy was constructed by incorporating a biocompatible hydrogel immersed in DW, followed by in situ mineralization of hydroxyapatite nanocrystals (HAps). This resulted in the preparation of a rigid osteoconductive composite hydrogel material with anisotropic and high strength. The incorporation of Hap nanofillers into the composite hydrogel resulted in a tensile strength of 67.8 MPa and an elastic modulus of 670.0 MPa. Furthermore, the Hap nanofillers were coupled with the biocompatible composite hydrogel to promote in vitro bone differentiation and in vivo bone tissue formation [27].

Bacterial cellulose (BC) is primarily manufactured through the in situ molding of bacteria in an aqueous medium containing glycogen. Bacterial cellulose (BC) is most commonly produced by *Komagataeibacter xylinus*, which is capable of producing a high yield of BC. BC has no functional groups other than the hydroxyl group (carboxyl group, carbonyl group, etc.). *Komagataeibacter xylinus* (formerly *Acetobacter xylococcus* or *Gluconacetobacter xylinus*) has attracted significant scholarly interest due to its ability to produce a wide range of utilizable glycogen species and relatively high BC production yields [28,29]. As demonstrated in a recent study by Zhang et al., a strain was engineered with a BC yield of 4.62 g/L, which was 48% higher than that of the wild type, by modulating the interactions between c-di-GMP metabolic proteins and combinatorially knocking out GE genes that could enhance BC production after knockdown [30]. Furthermore, a plethora of methodologies, including gene-editing technology, enzymatic hydrolysis substrate cultivation, and the utilization of agricultural by-products such as coconut water and corn stover as culture media, have been developed to enhance the yield and reduce the cost of BC production by *Komagataeibacter xylinus* [31,32,33,34,35]. Nevertheless, the challenges posed by genetic mutations in the strain itself, the instability of bacterial cellulose production, and the significant cost of cultivating the strain persist, thereby significantly restricting the large-scale application and commercialization of BC. The natural BC crystal structure is similar to that of α-type cellulose, while it has a high degree of polymerization (3000–9000) and a high degree of crystallinity (80–90%), and is hydrophilic. It is non-toxic and biodegradable, and has other beneficial characteristics. Furthermore, it retains excellent elasticity and flexibility in humid environments, making it an ideal material for biomedical applications. It is an optimal material for biomedical applications, including wound dressings, artificial corneas, drug carriers, and artificial bone tissues [36]. The BC/dextran composite hydrogel developed by Lin et al. for use in bioactive wound dressings not only exhibits the requisite mechanical properties for such applications, but also provides an optimal environment for wound healing through its high water content. The results of the in vivo experiments demonstrated its ability to prevent pathogen infection, it and also exhibited a superior capacity to induce cell proliferation and promote wound healing. The utilization of conductive materials facilitates the provision of enhanced electrical signals, which can be employed for the monitoring of wound healing and drug delivery processes [37]. Shi et al. enhanced the electroactivity of the BC hydrogel by incorporating polyaniline, demonstrating favorable biocompatibility with human umbilical vein endothelial cells in their experimental results. This innovative application has great potential both for producing raw materials and for use in the field [38].

Nanocellulose is the form of cellulose that exists at the nanometer scale, and the main types include cellulose nanocrystals (CNCs), cellulose nanofibers (CNFs), and bacterial nanocellulose (BNC) [39]. Cellulose chains are tightly stacked to form dense crystalline regions and are randomly entangled in disordered regions to form amorphous regions, which are susceptible to acid degradation. Therefore, CNCs are mainly produced by hydrolyzing the amorphous zone using cellulose under appropriate acid concentration and temperature conditions, resulting in short and rigid rods of CNCs in the crystalline zone, or by oxidation. CNF is a long and flexible nanofibrillar cellulose obtained by mechanical protofibrillation, and structurally consists of crystalline and amorphous zones together. BNC is assembled by biotechnology [40]. BNC has unique properties such as high purity, fine fibrillar structure, and excellent mechanical strength, which make it an ideal candidate for various applications in electronics.

Nanocellulose has many excellent properties and is becoming an ideal choice for multifunctional composites. Its main properties are (1) high mechanical strength: with a Young’s modulus of 100–130 GPa, it can be used as a reinforcing agent and is highly competitive in the production of functional materials with both flexibility and mechanical strength, such as films, gels, paper, electrodes, and capacitors; (2) high specific surface area and multiple active sites: the high specific surface area provides a structural basis for the porous composites to achieve nanopore structure, and the multiple hydroxyl reactive sites are also conducive to the modification of nanocellulose, realizing the diversity of composite material performance combinations and facilitating the adaptation of different material construction strategies; (3) excellent thermal stability and thermal expansion: the thermal decomposition temperature of nanocellulose is 200–300 °C, and the coefficient of thermal expansion is about 10^−7^K^−1^, which is an excellent choice for the fabrication of electronic devices such as flexible electrodes and supercapacitors, i.e., the structure and performance of the device can still be stable when the device temperature rises after a long period of use; and (4) high biocompatibility: it can be used as the main carrier for drug delivery, implantable electronic devices, artificial bones, and other biomedical materials. Meanwhile, properties such as high potential window width, easy processing, degradability, and dispersibility in inorganic materials demonstrate the strong competitiveness of nanocellulose in the field of advanced multifunctional materials applications [41,42]. Sun et al. constructed a composite electrode CNC–AgNW/PDA-PDMS using silver nanowires, cellulose nanocrystals, poly(dopamine), and poly(dimethylsiloxane). The electrode not only possesses surface smoothness, high transparency, strong antioxidant properties, and low thin film resistance (about 11 Ωsq^−1^), but also tensile stability and fatigue resistance after forming an EL device with a light-emitting layer. The EL device also exhibits excellent brightness, flexibility, and biocompatibility when used under water and at extreme temperatures [43]. Wei et al. prepared a conductive hydrogel by combining cellulose nanofibers and template carbon nanotubes consisting of hybrids (CNF/CNT nanohybrids) as the reinforcing conductive backbone with acrylamide. The hydrogel achieved a tensile strength of about 119.2 kPa and an electronic conductivity of about 2.7 mS/cm, while maintaining good flexibility at low temperatures (<−24 °C) and good long-term water retention in open environments (>10 d) [44].

Modified cellulose, while retaining the original chemical, mechanical strength, and biocompatibility properties, can be creatively modified by surface modification to have more application possibilities. Nowadays, various modification strategies have been found, such as oxidation reaction, esterification reaction, etherification reaction, DA reaction, alkyne–azide reaction, and light-induced thiol–alkene reaction [45,46,47,48,49,50]. Through the above modification strategies, cellulose can be modified to have more functional groups, which provides the possibility to meet various needs. For example, Guo et al. prepared a dual-network (DN) conductive hydrogel by combining sodium carboxymethyl cellulose and polyacrylamide under the condition of DMSO–H_2_O as a solvent. This conductive hydrogel maintains an ionic conductivity of about 1.07 mS/cm, while possessing high tensile strength, toughness, transparency, and antifreeze properties, which is very promising for applications in the field of flexible optical and electrical components [51].

**Figure 2:**
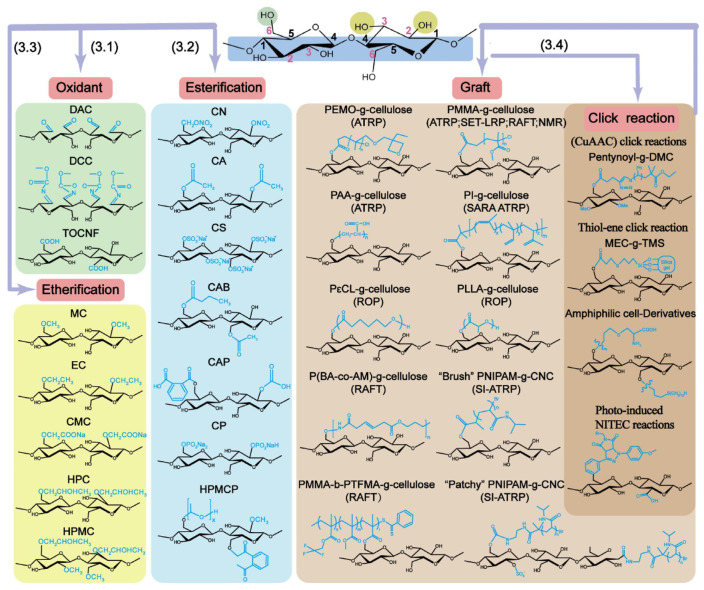
The most common modifications of cellulose [24]. Copyright 2023, ELSEVIER.

In order to more effectively address the requirements of practical applications, the production of cellulose raw materials often needs to be combined with a variety of materials to have a greater value of modification. The most common modification of cellulose is illustrated in the accompanying Figure 2.

## 3. Cellulose-Based Conductive Hydrogels

### 3.1. Overview of Cellulose-Based Hydrogels

#### 3.1.1. Overview of Hydrogels

Hydrogel is a functional polymer material with a three-dimensional network structure formed by hydrophilic polymer chains through a process of cross-linking, whereby hydrogen bonding, van der Waals force, covalent bonding, ligand bonding, host–guest interaction, electrostatic effect, and physical entanglement combine to create a stable and malleable structure. Hydrogel contains hydrophilic groups, including hydroxyl, carboxyl, and amide groups, among others. The dissolution of hydrogel is contingent upon the absorption of water. Once this occurs, the gel retains its shape and composition, exhibiting properties analogous to those of the extracellular matrix [52]. The high water content and retention capacity enable hydrogel to absorb and retain a substantial volume of water or biological fluid. The cytocompatibility, ease of processing, and multifunctionality of hydrogels demonstrate excellent potential for application in the biomedical field, including the provision of wetting conditions for wound healing, the development of tunable mechanical properties for artificial tissues, and the facilitation of electrical signal conduction for wearable or implantable devices [53]. With the ongoing advancement of properties such as self-healing, strong adhesion, self-powering, and multi-environmental responsiveness, hydrogels are increasingly demonstrating the potential to become intelligent advanced materials.

With the increasingly serious problem of environmental pollution and the scarcity of non-renewable resources, natural polymer hydrogels have received widespread attention. Among them, cellulose, the richest biomass resource on earth, is very competitive as a synthetic raw material of hydrogel, and cellulose-based hydrogel has shown a wide range of application prospects and has already yielded promising results in the fields of medicine, environment, agriculture, electronics, and so on [54].

#### 3.1.2. Structure of Cellulose-Based Hydrogel Networks

**Figure 3:**
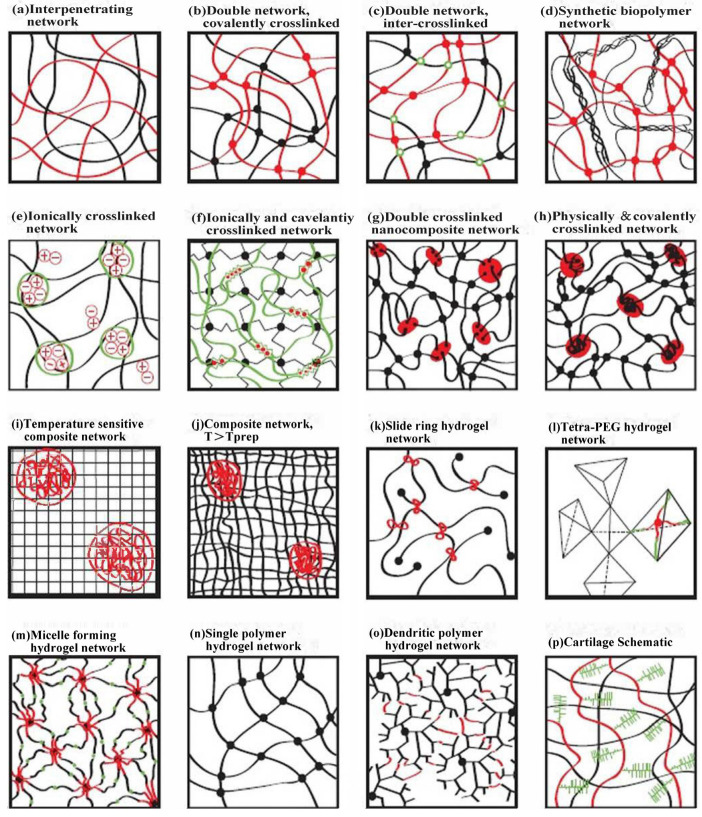
Common hydrogel cross-linked network structures. Intuitive representation of the covalent or non-covalent forms of cross-linking in a 3D hydrogel network [55]. Copyright 2013, SPRINGER NATURE.

The synthesis of hydrogel can be broadly categorized into two main types: covalent cross-linking and non-covalent cross-linking. Conventional hydrogel, which is composed of a solely covalent bonding network structure, is inherently limited in its practicality and prospective applications due to the constraints imposed by its mechanical strength, flexibility, and other intrinsic properties. In order to enhance the applicability of hydrogel in real-world scenarios, novel hydrogel is typically designed to comprise two or more distinct covalent or non-covalent bonding mechanisms, which collectively facilitate the formation of a single polymer network or multiple. Covalent cross-linking primarily employs cross-linking agents or irradiation to facilitate covalent bonding interactions, whereas non-covalent cross-linking predominantly relies on interactions such as physical entanglement, hydrogen bonding, and electrostatic interactions. As illustrated in Figure 3, the combination of covalent and non-covalent bonds results in the formation of a diverse range of polymer network structures. These include interpenetrating network structures, dual/multiple network covalent cross-linking structures, ionic cross-linking network structures, and others. Cellulose-based hydrogels exhibit analogous applications in network structure. The following section will present a selection of cellulose-based hydrogels, predominantly from the three perspectives of single network structure, interpenetrating network structure, and semi-interpenetrating network structure [55].

The raw materials employed in the synthesis of single-network cellulose-based hydrogels are primarily cellulose and cellulose derivatives, with a single polymer component. However, the system exhibits a range of potential cross-linking modes, including single or multiple options. Yang et al. prepared a single-network cellulose-based hydrogel by using sodium hydroxide/urea/water as a solvent for the solubilization of 2% flax cellulose and ethylene glycol diglycidyl ether (EGDGE) as a cross-linking agent. The hydrogel exhibits a compressive strength of approximately 54.48 kPa and an equilibrium swelling ratio of approximately 38 g/g. Immersion in varying concentrations of acetic acid solution allows for the generation of pore structures with differing sizes, with the swelling ratio reaching 49 g/g [56]. However, the compressive strength declines with increasing acid concentration. Similarly, higher-strength cellulose-based hydrogels were prepared using a dual cross-linking strategy that combined physical and chemical methods by Zhao et al. In addition to using alkali/urea/water as a solvent to dissolve cellulose and ECH as a cross-linking agent, physical cross-linking was induced by the addition of ethanol, which formed hydrogen bonds with the entanglement of molecular chains. The cellulose hydrogel exhibited a tensile strength of 2.7 MPa and an elongation at break of 81%, thereby demonstrating the successful reinforcement of the hydrogel [57]. In the case of new hydrogel materials, single-network hydrogels have the advantage of easy preparation and control; however, their relatively weak mechanical properties represent a significant disadvantage that limits their practical use. For example, Jeong et al. prepared a single-network cellulose-based hydrogel using CMC and ECH, but the compressive strength was only 37.4 Pa in the mechanical test [58]. The construction of different hydrogel network structures through the use of different synthesis strategies and monomer selection represents a potential avenue for improving performance.

Interpenetrating network cellulose-based hydrogels (IPNs) are characterized by an interpenetrating network structure, comprising two or more polymer networks formed by individual cross-linking. These polymer networks are distinct and do not interconnect with each other [59]. The synthesis of interpenetrating network hydrogels is achieved through two principal methodologies: (1) The stepwise polymerization method: the initial polymer network is prepared and subsequently immersed in a solution system comprising monomers, initiators, and cross-linking agents of a second polymer network, which enables the second polymer network to be polymerized within the first, thereby forming an interpenetrating network structure, and (2) the synchronous polymerization method: in the same reaction vessel, a variety of monomers can be added according to their respective polymerization and cross-linking systems, which then facilitate the formation of a synchronous interpenetrating network structure [60]. A dual-network hydrogel is a special interpenetrating network structure comprising a highly cross-linked primary network and a loosely cross-linked secondary network [61]. Wang et al. employed a step-by-step polymerization method to synthesis the dual-network structure of the hydrogel, comprising BC and silk proteins (SF). In addition to exhibiting high mechanical strength and biocompatibility, the BC/SF hydrogel displays a compressive strength of 55.21% strain with 1.49 MPa, which is superior to that of both the pure BC hydrogel (1.1 MPa) and the pure SF hydrogel (0.1 MPa) [62]. Song et al. prepared a dual-network hydrogel by synchronous polymerization. The yield stress of the CMC hydrogel was 19.50 kPa, but the introduction of PEI and the formation of the dual-network structure greatly enhanced the mechanical strength. The CMC/PEI exhibited a yield stress of 77.88 kPa and a compressive strength of 0.5 kPa. The yield stress was observed to be 77.88 kPa [63]. An IPN hydrogel exhibits a more stable cross-linking structure, enabling it to withstand mechanical loss during deformation. It displays enhanced strength and toughness, and has a broader range of potential applications [64,65]. Concurrently, the stepwise polymerization method allows for more precise regulation of IPN hydrogels. In contrast, the synchronous polymerization method is more cost-effective and time-efficient. Furthermore, the selection or construction of an optimal synthetic pathway can significantly enhance the commercial viability of IPN.

Semi-interpenetrating network cellulose-based hydrogels have a semi-interpenetrating network structure (semi-IPN), which consists of multiple interpenetrating polymer networks, but at least one of the polymer networks is in a non-cross-linked form, such as a linear or branched chain [66]. Semi-interpenetrating network hydrogels are mainly formed by selective cross-linking of linear polymer chains in a mixed system containing all types of polymers, monomers, initiators, and cross-linkers [67]. Cellulose/polyurethane semi-interpenetrating network hydrogels were synthesized by Cai et al. The cellulose/polyurethane semi-interpenetrating network hydrogel had a tensile strength of 160 MPa, an elongation at break of 6%, and a Young’s modulus of about 5.7 GPa. However, the pure cellulose hydrogel only had a tensile strength of 110 MPa, an elongation at break of 4.5%, and a Young’s modulus of 4.2 GPa [68]. In addition to improving the mechanical properties, Gomez et al. synthesized a semi-interpenetrating network hydrogel by adding DVS to a hybrid system of HEC and CMC. The semi-interpenetrating network structure of this hydrogel consists of uncross-linked CMC and cross-linked HEC, and the uncross-linked CMC gives the hydrogel better water retention while increasing the free Na^+^ ions, which improves the ionic conductivity [69]. The semi-interpenetrating network structure can better improve the performance of hydrogel. Firstly, it can improve the mechanical properties of the hydrogel, due to the similar enhancement mechanism observed with interpenetrating network structures, which also utilize linear polymer chains as reinforcing phases [70]. Secondly, the introduction of ions or other groups can facilitate better electrical conductivity, adhesion, and sensitivity to stimuli in the hydrogel [71]. Thirdly, the semi-interpenetrating network structure allows for a higher porosity, which in turn results in more mobile cross-linked networks in the chain segments [72]. Hydrogels based on cellulose with a semi-interpenetrating network structure are more functional and facilitate the broader application of cellulose-based hydrogels in diverse fields.

Cellulose-based hydrogel exhibits a range of properties, including the capacity to absorb and retain water, as well as biocompatibility, environmental tolerance, and degradability. Additionally, it can be derived from diverse cellulose sources, modified, and processed to impart unique characteristics such as electrical conductivity, antimicrobial properties, and robust mechanical strength [73]. To further promote the application of cellulose-based hydrogel in real life and facilitate the development of functionality and intelligence, the construction of cellulose-based conductive hydrogel is of particular importance. The following section will present a detailed account of the construction and practical applications of cellulose-based conductive hydrogels.

### 3.2. Construction of Cellulose-Based Conductive Hydrogels

The above not only describes the application advantages of cellulose as a biomass material in terms of low price, environmental protection and renewability, richness in variety, functional diversity, etc., but is also very competitive and promising in terms of its application as a hydrogel material. However, in order to better meet the development needs of the Internet of Everything and artificial intelligence, combined with the excellent characteristics of cellulose itself, the development of cellulose-based conductive hydrogel will shine in the field of flexible electrodes, intelligent medicine, and others.

#### 3.2.1. Conductive Forms of Cellulose-Based Conductive Hydrogels

**Figure 4:**
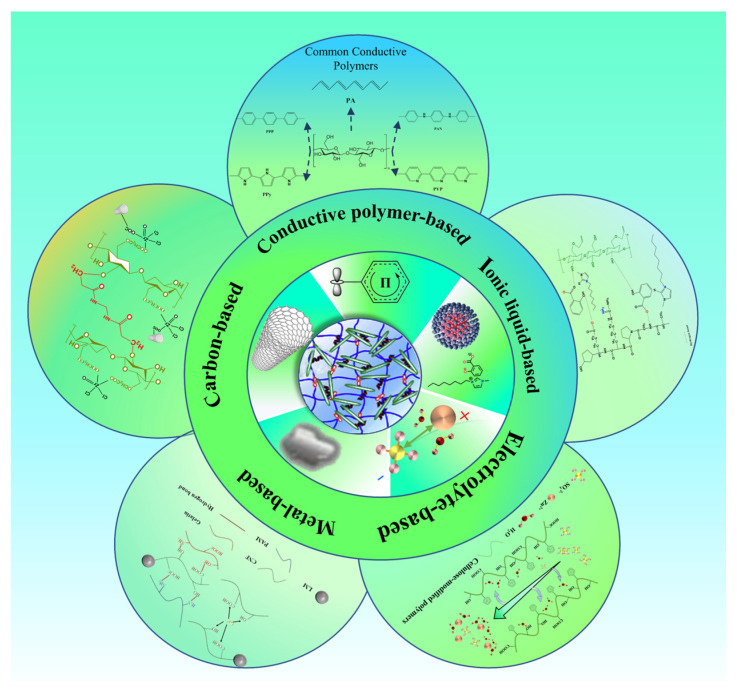
Classification of conductive cellulose-based hydrogels. Metals, carbon, and conductive polymers form electrically conductive hydrogels. Electrolyte and ionic liquid groups form ionic conductive hydrogels.

The conductive capacity of hydrogel is frequently contingent upon the incorporation of conductive fillers and ions. In accordance with the distinctions in conductivity, hydrogel can be classified into two principal categories: electronic conductive hydrogel and ionic conductive hydrogel [74]. Cellulose itself does not possess the ability to conduct electricity. However, it is relatively straightforward to modify cellulose chemically, and it has numerous active sites, high mechanical properties, and other characteristics that can be combined with other conductive substances to create a variety of forms of cellulose-based conductive hydrogel [75]. To illustrate, cellulose can be employed to construct a gel network, thereby creating conductive hydrogels. Xiao et al. utilized phenylboronic acid-modified cellulose nanocrystals (CNC–ABAs) and subsequently grafted them with multiwalled carbon nanotubes (MWCNTs) on polyvinyl alcohol (PVA) to generate a conductive hydrogel with a double-cross-linked network and superior mechanical properties. The combination of MWCNTs and NaOH resulted in a conductivity of approximately 3.8 × 10^−2^ S/m, which is indicative of enhanced electrical conductivity. The experimental conditions exhibited a tensile strength of 227.0 kPa, a strain capacity of 395.0%, and a modulus of elasticity of 9.0 kPa, and demonstrated good biocompatibility and self-healing properties [76]. Cellulose can also be employed as a reinforcing agent to create conductive hydrogels. Huang et al. prepared cellulose-based conductive hydrogels by blending powdered cellulose and polyacrylamide in a straightforward one-pot method. The formation of cross-links between hydrogel chains is facilitated by a variety of forces, including hydrogen bonding and ionic coordination bonding. This leads to the development of a network structure with enhanced mechanical properties, including a tensile strength of 2795% and a tensile stress of 12,806 kPa. Additionally, the hydrogels exhibit excellent fatigue resistance and self-healing properties [77]. Subsequently, an array of cellulose-based conductive hydrogels will be presented, classified according to their conductive form (Figure 4).

Electronically Conductive Cellulose-Based Hydrogels

Electron-conducting hydrogels are mainly used to realize the transfer of electrons by using conductive fillers (metallic nanomaterials, carbon-based conductive materials, and conductive polymers) to construct a conductive pathway in the gel matrix [78].

Metal-Based Cellulose Conductive Hydrogels

Metal nanomaterials are a preferred option for the synthesis of conductive hydrogels, offering a combination of high electrical conductivity inherent to metal materials and the unique properties of nanomaterials, including surface effects (reactivity and catalytic activity) and small-size effects (differences in light, heat, magnetism, etc.). Metal nanomaterials are frequently utilized in the form of metal nanoparticles, metal nanorods, and metal nanowires, among other configurations [79]. Lin et al. modified cellulose nanocrystals (CNCs) to obtain Ag/TA@CNC, and subsequently synthesized a conductive hydrogel, PB-Ag/TA@CNC, comprising a dynamic boronate bonding cross-linked network with polyvinyl alcohol (PVA) and borate trihydrate (B(OH)_3_). The dynamic cross-linked structure and Ag/TA@CNC imparted excellent mechanical properties to the hydrogel, including a tensile strength of up to 98.6% and a self-healing capacity exceeding 4000% within 10 min. As the concentration of Ag/TA@CNC increased from 0 to 5 wt%, the conductivity rose from 0.16 S/m to 4.61 S/m. The addition of the CNC-modified functional filler resulted in the hydrogel exhibiting excellent adhesion and antimicrobial properties. The PB-Ag/TA@CNC hydrogel was successfully assembled into a self-healing flexible sensor with a wide range of strain-sensing capabilities, making it an ideal candidate for motion monitoring. Furthermore, the feasibility of PB-Ag/TA@CNC was also confirmed for use in e-skin and touch screen applications [80]. Liquid metal (LM), which is soft and malleable at room temperature, has also been employed extensively in the field of conductive materials. Zou et al. employed liquid metal (LM) stabilized by carbon nanofiber (CNF) to initiate the polymerization of acrylamide. Subsequently, reduced graphene oxide (rGO) was utilized to bridge the minute droplets of LM, facilitating further cross-linking under glycerol conditions. This resulted in the formation of a composite hydrogel, CNF@LM/polyacrylamide/rGO/gelatin/glycerol (CLPRGG). The hydrogel displayed remarkable characteristics, including high stretchability (>1317%), high environmental adaptability (−80~80 °C), robust adhesion, electromagnetic shielding, and multifunctional sensing capabilities. The electrical conductivity of the CLPRGG hydrogel was observed to increase in conjunction with an increase in the LM content. The presence of rGO was also found to enhance the formation of conductive channels, thereby significantly improving the electrical conductivity of the hydrogel. CLPRGG has been successfully employed for temperature monitoring and strain sensing, exhibiting high sensitivity and repeatability for the stable monitoring of changes in electrical signals. Additionally, its unique EMI-shielding properties hold considerable promise for further special applications [81].

Carbon-Based Cellulose Conductive Hydrogels

Carbon-based conductive materials are highly competitive in the field of constructing conductive hydrogels due to a number of advantageous properties, including improved mechanical properties, a large specific surface area, environmental and chemical stability, and an inexpensive price. Additionally, these materials facilitate high conductivity through π-conjugated conductive channels, enabling electron transfer within the hydrogel. The most commonly utilized carbon-based conductive materials include graphene (GN), carbon nanotubes (CNTs), carbon black (CB), and two-dimensional transition metal carbide (MXene), among others [82]. The purity and graphitization of carbide have been observed to affect the off-domain electrons, and consequently the conductive properties. As an illustration, Zheng et al. prepared self-healing conductive hydrogels by homogeneously dispersing TOCNF–GN nanocomposites in PAA. The finally synthesized TOCNF–GN/PAA composite hydrogel has good mechanical properties, exhibiting 2.54 MPa compressive strength, 0.32 MPa tensile strength, 850% tensile strength, and self-healing properties (12 h, 96.7%) in the test. The material exhibits a conductivity of approximately 2.5 S/m, which makes it suitable for use as a highly sensitive strain sensor (GF = 5.8). This suggests that it has significant potential for integration into wearable flexible devices in the future. The two-dimensional material MXene, which exhibits favorable conductive and electrochemical energy storage properties, coupled with distinctive mechanical, magnetic, and nanostructural characteristics, represents an optimal option for the fabrication of cellulose-based conductive hydrogels [83]. As reported by Yin et al., MXene was produced by mixing Ti_3_AlC_2_, LiF, and HCl, namely multilayer Ti_3_C_2_TX, and subsequently, a PAA/SCMC/Ti_3_C_2_TX hydrogel was prepared by employing the one-pot method in conjunction with carboxymethyl cellulose (SCMC). The hydrogel exhibits a conductivity of (1.09 ± 0.12) S·m^−1^, and the combination of excellent mechanical properties and toughness makes it suitable for use in the fabrication of sensitive strain sensors and temperature sensors. Furthermore, the supercapacitor composed of the hydrogel displays considerable potential for application. This represents a novel solution for the construction of flexible electronic devices [84].

Conductive Polymer-Based Cellulose Conductive Hydrogels

Conducting polymers are long-chain polymers with a conjugated structure, denoted by ICP, which construct electronic pathways primarily through the free movement of electrons along the polymer backbone. This is achieved by the off-domain π electrons on the unsaturated backbone [85]. Since the year 2000, when the Nobel Prize in Chemistry was bestowed upon three scientists, McDermid, Hegger, and Hideki Shirakawa, for their research on conductive polymers, the utilization of these polymers in the preparation of conductive hydrogels has become a common practice. This is due to several key advantages, including superior dispersion compared to nanomaterials, a straightforward synthesis and processing method, high and easily adjustable electrical conductivity, and excellent flexibility. Polyacetylene (PA), polypyrrole (PPy), polythiophene (PT), polyaniline (PANI), poly(3,4-ethylenedioxythiophene) (PEDOT), and other conductive polymer materials endow hydrogel materials with conductivity while simultaneously enhancing their properties, such as ductility and mechanical strength. The construction of conductive polymer-based cellulose conductive hydrogels is also principally conducted through two methods: direct mixing of conductive polymers with cellulose-based hydrogel components and in situ polymerization of conductive polymer monomers within the cellulose skeleton or gel matrix [86,87]. Suneetha et al. subjected CMC and dopamine hydrochloride (DA) to a coupling reaction to produce CMC-DA, which was then dissolved in double-distilled water (DDW). Subsequently, BX, AM, and MBA were added sequentially, and finally APS and TAMED were added to react for 3 h to obtain a self-healing conductive hydrogel, CDB-PAM-PEDOT: PSS, with high tensile properties and tissue adhesion. The addition of PEDOT: PSS resulted in the homogeneous dispersion of the latter in the CMC-DA solution, thereby significantly enhancing the properties of the hydrogel. The CDB-PAM hydrogel exhibited tensile properties (2763%) and tensile strength (36.9 ± 2.3 kPa), while the incorporation of 2.0 wt% PEDOT: PSS led to an increase in tensile properties (2873%) and tensile stress (44.9 ± 2.6 kPa), and a reduction in elastic modulus (3.10 ± 0.15 kPa). The long-term reusable adhesion (65.1 ± 2.2 kPa for adhesion to pig skin) was ensured by maintaining the catechol groups in the hydrogel. In addition to exhibiting excellent biocompatibility, the PEDOT present in the hydrogel demonstrated excellent electrical conductivity (41.6 S/m), with the catechol/quinone moiety of CMC-DA serving as a pathway for charge transfer [88].

Ionically Conductive Cellulose-Based Hydrogels

The porous structure of the hydrogel network is a key benefit of hydrogels. The internal pore structure is capable of capturing and storing a substantial quantity of water, thereby providing a structural basis for the free movement of ions and maintaining high mobility. This results in the formation of an ionic conductive hydrogel with conductive properties [89]. In comparison to electronically conductive hydrogels, ionic conductive hydrogels exhibit superior characteristics, including enhanced transparency, improved mobility, and simplified processing and synthesis. The introduction of ions in hydrogels also relies on electrolytes and ionic liquids.

Electrolyte-Based Cellulose Conductive Hydrogels

Electrolyte-based conductive hydrogel is primarily composed of soluble inorganic salts (NaCl, LiCl, CaCl_2_, etc.) and acids (HCl, boric acid, phytic acid, etc.), which are introduced directly into the hydrogel’s three-dimensional network [90]. The free ions can be divided into two main forms: physical doping and chemical doping. In the case of physical doping, the free ions play a conductive role in charge transfer without bonding to generate a form of doping. In contrast, in the case of chemical doping, the free ions conduct while simultaneously forming ligand bonding centers, which in turn form different forms of hydrogel network structures [91,92]. Furthermore, an electrolyte monomer can be prepared into a polyelectrolyte-based hydrogel with good conductivity through the polymerization of a polyelectrolyte. Polyelectrolytes are typically synthesized from charged monomers, including sodium methacrylate, 2-acrylamido-2-methylpropanesulfonic acid (AMPS), and 2-methacryloyloxyethyltrimethylammonium chloride (DMC) [93]. Polyelectrolytes encompass polycationic electrolytes, polyanionic electrolytes, and polyamphoteric electrolytes, which are electrically conductive due to the generation of free ions through the ionization of ionized groups. In a conventional approach, Yu et al. immersed CNF in a CaCl_2_/sorbitol solution to obtain a CS-NC organogel. The presence of sorbitol and CaCl_2_ in the hydrogel endowed it with high ionic conductivity, antifreeze properties, and water retention ability. The strong hydrogen bonding between sorbitol and water molecules is the primary factor contributing to the material’s exceptional low-temperature resistance (down to −50 °C) and remarkable water retention (over 90% weight retention). This is due to the strong hydrogen bonding preventing the formation of merging crystals and the evaporation of water. Additionally, the CNF plays a crucial role in enhancing the material’s mechanical properties [94].

Ionic Liquid-Based Cellulose Conductive Hydrogels

Ionic liquid-based conductive hydrogels are ionic conductive gels that exhibit high ionic conductivity and high thermal stability. They are prepared using ionic liquids as the dispersion medium. An ionic liquid (IL) is a combination of organic cations (e.g., ammonium, sulfonium, phosphonium, imidazole, triazole, pyridinium, pyrazolium, etc.) and organic anions (e.g., borates, halogenated substances, etc.). These are mostly molten salts with a melting point of less than 100 °C [95]. The asymmetric and bulky nature of the ionic structures that comprise ionic liquids results in reduced interactions between anions and cations, in comparison to the tightly stacked, traditional symmetric ion pairs. This phenomenon is the underlying cause of the lower melting point observed in ionic liquids. In addition to its properties of high ionic conductivity, high thermal stability, wide electrochemical window, and nonvolatility, IL can be developed into an anhydrous conductive gel, thereby avoiding the drawbacks associated with dehydration and freezing cracking [96]. Furthermore, it represents an excellent choice for the preparation of conductive gels as an electrolyte. In a study conducted by Lee et al., cellulose nanocrystal networks were employed to enhance ionic liquids, resulting in the development of CNC/PIL ionic gels that exhibited shape persistence and high conductivity. The combination of needle-shaped CNCs with PIL resulted in the formation of a network structure comprising multiple hydrogen bonds and electrostatic interactions. This structure was capable of confining a significant quantity of ionic liquid (95 wt%) while maintaining the shape of the individual components. The resulting conductive gel exhibits excellent mechanical properties and a high compressive modulus of elasticity (5.6 MPa), while maintaining the high electrical conductivity of the original ionic liquid (7.8 mS/cm^−1^) [97]. Further utilizing ionic liquids, Zhu et al. employed ionic liquids to co-synthesize a PACxVy hydrogel with properties including high elasticity, hyper-extensibility, and low hysteresis. This was achieved by combining 1-vinyl-3-butylimidazole (VBIMBr, ILs) with AM and CNF. The hydrogel is an effective solution that meets the challenging requirements of elastic wound dressings and flexible epidermal bioelectronics, while also offering antimicrobial properties, promotion of tissue regeneration, and high electrical conductivity (2.47 S/m). This enables the accurate capture of motion signals, ranging from large strains to small physiological signals, such as body movement, heart rate, pulse, and body temperature. In conjunction with a bespoke single-chip design, this offers the potential for the development of innovative solutions to enhance individualized therapy and health management [98].

#### 3.2.2. Performance Requirements for Cellulose-Based Conductive Hydrogels

Lightweight, flexibility, efficiency, and multifunctionality are the conditions necessary for flexible electronic products to play a more important role in life. Cellulose-based conductive hydrogel-constructed flexible electrodes, soft robots, electronic skin, and a series of new flexible electronic products will play a broader role in the future, but relying on the real-life requirements of practical applications, there are still many properties that need to be improved and popularized. Next, we will present some typical requirements as an example and illustration:

(1) Cost, process, and other industrialization needs: Cost regulation, simple and environmentally friendly synthesis methods, and intelligent production and manufacturing processes can significantly facilitate the large-scale application of cellulose-based conductive hydrogel in the field of flexible electronics. Cellulose itself is a renewable resource with abundant content and high annual production; thus, the cost is readily manageable. The simple synthesis method, exemplified by the one-pot approach, is conducive to the large-scale production of cellulose-based conductive hydrogels. For instance, Li et al. employed the one-pot method to generate ionic conductive hydrogels, namely PVA–CNF organogels, which exhibit remarkable stretchability (696%), rapid response (130 mS), extensive operational temperature range (−50 °C to 50 °C), and exceptional long-term stability (30 days) [99]. The selection of green raw materials and synthesis processes is also a prevalent trend in contemporary research. For instance, Zhang et al. employed a natural antifreeze mechanism, cotton liner pulp as a raw material, and a ZnCl_2_/CaCl_2_ aqueous solution dissolved in cotton cellulose to create a cellulose-based ion-conducting hydrogel with an operational temperature range of −70 °C to 50 °C. This hydrogel exhibited mechanical stability and self-healing capabilities [100]. In order to better align with the prevailing trends, the integration of automated and intelligent industrial production methodologies is also of particular significance. Giachini et al. have demonstrated the successful combination of materials engineering and digital processing technology in the fabrication of functional gradient materials (FGMs), which have a multitude of potential applications, including biomedical and architectural domains. The specific scheme is to design cellulose-derivative solutions with the objective of achieving tunable viscoelasticity and controllable extensibility. In parallel, a digital workflow is being developed that embeds the required gradient information and generates customized G-codes with the purpose of controlling the extrusion and positioning systems of three-dimensional (3D) printers and syringe pumps. The integrated application of these physical and mathematical techniques provides a means of generating multidirectional and continuous stiffness gradients in a manner that is compatible with the intrinsic properties of cellulose, thereby offering a potential avenue for utilization in a diverse range of fields, including tissue engineering. This intelligent production process serves as an exemplar for the industrialization of cellulose-based conductive hydrogels [101]. The coordination of cost control, the implementation of simple and environmentally friendly synthesis methods, and the utilization of intelligent production techniques, in addition to other practical industrial production requirements, will facilitate the realization of cellulose-based conductive hydrogels in people’s daily lives, enabling them to play an increasingly important role.

(2) Regulation of mechanical properties: Hydrogel remains a predominantly malleable and brittle material. To better align with the demands of practical applications, cellulose-based conductive hydrogel can be reinforced through physical or chemical means to enhance its mechanical properties. Nevertheless, the unceasing enhancement in mechanical strength does not necessarily guarantee enhanced performance. The requisite mechanical strength must be calibrated in accordance with the specific application scenarios and purposes. Accordingly, a straightforward and expedient approach to modulating the mechanical properties is essential to expand the potential applications of cellulose-based conductive hydrogels. Zhang et al. employed a LiOH/urea alkaline solution to dissolve the BC suspension, followed by the use of ECH for cross-linking, stretching, mineralization, and other processes to generate robust, stable, ion-conductive bacterial cellulose hydrogels (ABCHs). The urea content can be employed as a means of regulating the hydrogel properties. The tensile strength and strain of the hydrogel can be regulated by controlling the urea content [102]. This straightforward and accessible method for controlling mechanical properties offers insights into the potential for regulating industrial production processes. Three-dimensional printing can fabricate cellulose-based hydrogels with customized internal architectures. For example, lattice-like or honeycomb structures can be printed to enhance the mechanical integrity of the hydrogel while maintaining its flexibility.

(3) Sensitive response to environmental stimuli: The sensitive environmental response capability of hydrogel makes it a valuable material for use in sensors, biomimetic devices, environmental monitoring, and other applications where regulatory capabilities and response diversity are required [103]. The combination of cellulose as a raw material with autogenous characteristics allows for the preparation of environmentally stimulus-responsive cellulose-based conductive hydrogel, which can play an irreplaceable role in a multitude of fields.

Depending on the source of the stimulus response, they can be categorized as follows: physical stimulus-responsive (e.g., light, temperature, mechanical stress, etc.), chemical stimulus-responsive (e.g., pH, ions, etc.), and biostimulus-responsive (e.g., enzymes, glucose, etc.). For example, the CMC/PEO hydrogels prepared by Kanafi et al. exhibited excellent pH responsiveness in the presence of carboxymethylcellulose, which has significant potential for use in drug release control [104]. Oechsle et al. also employed a straightforward preparation method to obtain CO_2_-switchable CNC hydrogels, which demonstrated CO_2_ stimulus responsiveness without the necessity of the functionalization of CNCs [105]. The potential for cellulose-based conductive hydrogels to be applied in a smart manner is further enhanced if they can be combined with a signaling function, thereby enabling the integration of multiple environmental stimulus responses and the capture of stimulus changes and signaling with greater sensitivity.

(4) Biocompatibility: An important performance aspect for biomedical applications is that 3D-printing enables the incorporation of bioactive molecules or cells in a controlled manner. The printing process can be adjusted to encapsulate cells within the cellulose-based hydrogel, ensuring their viability and functionality. This precise control over the material composition at the micro-scale, achievable through 3D printing, is essential for meeting the biocompatibility requirements in tissue engineering and drug delivery applications.

(5) Self-healing: The capacity for self-healing represents a defining characteristic of conductive hydrogel-type flexible electronic devices, and is also a crucial performance indicator. The self-healing pathway of a polymer material can be broadly classified into two categories: exogenous self-healing and endogenous self-healing. Exogenous self-healing is dependent on the functionality of the loaded repair agent to achieve the objective of self-healing. However, the restricted quantity of the repair agent significantly constrains the repair capability and the practical deployment of conductive hydrogel. Endogenous self-healing is primarily achieved through reversible covalent and non-covalent bonding. The former encompasses acylhydrazone, disulfide, Diels–Alder, borate, imine, and reversible free radical reactions. Reversible non-covalent bonding encompasses hydrogen bonding, electrostatic interactions, metal coordination, and host–guest interactions, which represent the predominant application forms of self-healing conductive hydrogels in current practice [106]. These structural features are essential for the successful deployment of cellulose-based conductive hydrogels. As demonstrated by Lin et al., the preparation of PB-Ag/TA@CNC-conducting hydrogels exemplifies this approach. This conductive hydrogel employs the dynamics of dynamic borate and hydrogen bonding to endow this bionic conductive hydrogel with self-healing ability, reaching 98.6% in 10 min [80]. By ingeniously combining various available self-healing solutions, cellulose-based conductive hydrogels can be better endowed with self-healing ability, which also ensures the durability of the material and the stability of signal transmission, electrochemical properties, and other characteristics.

(6) Stable conductive properties: It is of great importance to ensure the stable output of conductivity in all types of electronic and intelligent applications in the field of essential performance requirements, regardless of whether the hydrogel in question is of an electronic conductive or ionic conductive nature. In this regard, cellulose-based conductive hydrogels offer a number of potential solutions to this problem. For example, Chen et al. prepared a B-PVA/NFC hydrogel with an ionic conductivity of 1.81 mS·cm^−1^ using NFC as the substrate. The assembled zinc ion supercapacitor not only exhibited a capacitance of 504.9 mF·cm^−2^ but also retained 95.3% of its capacity after 5000 cycles. This excellent cycling performance demonstrates the stability of the cellulose-based conductive hydrogel’s conductive properties [107]. Furthermore, Quan et al. obtained Sor-Cel gel electrolyte by modifying cellulose extracted from wheat straw by adding sorbitol. This resulted in a material with an excellent freezing-resistant ability and a wide operating temperature range, which satisfied the stable conductive ability under more extreme conditions. The material exhibited a conductivity of 35.4 mS/cm at 20 °Cand 19.7 mS/cm at −40 °C [108]. Three-dimensional printing can control the orientation and distribution of conductive additives, such as carbon nanotubes or metallic nanoparticles. By precisely depositing these materials layer by layer, we can create conductive pathways that optimize the electrical conductivity of the hydrogel, which is crucial for applications like flexible electronics.

The combination of favorable self-healing properties with good cycling stability and a wide operating temperature range enables stable conductivity under more extreme conditions. Nevertheless, it is also a significant objective in the advancement of cellulose-based conductive hydrogels to ascertain how to attain conductive hydrogel stability at more extreme conditions and to combine a variety of environmental stability abilities.

(7) Multifunctionality: The multifunctionality of cellulose-based conductive hydrogel composites represents a significant advantage; however, the challenge remains to coordinate the individual properties. In addition to the mechanical properties, biocompatibility, and degradability of such hydrogel materials, universal properties, electrical conductivity, self-healing, antimicrobial activity, cell proliferation, and stimulus response sensitivity are also essential for achieving stable multifunctionality. Proper regulation of these properties is necessary to ensure the optimal performance and reliability of the hydrogel materials.

The fulfilment of the aforementioned performance requirements will lead to a significant expansion in the applications and popularity of cellulose-based conductive hydrogels. Furthermore, this will facilitate the replacement of more traditional materials and electronic devices.

## 4. Applications of Cellulose-Based Conductive Hydrogels

**Figure 5:**
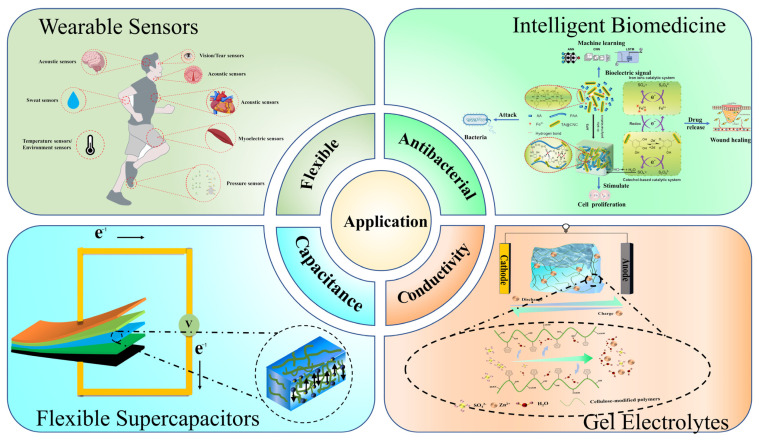
Applications of cellulose-based conductive hydrogels. As a wearable sensor, it can characterize important human physiological indicators such as acoustics, heartbeat, pressure, and environment through electrical signal visualization. Applied to intelligent biomedicine, such as the work of Li H et al. [109], conductive hydrogels enable antibacterial effects, cell regeneration, and wound healing while using changes in electrical signals to better facilitate smart applications. As a flexible supercapacitor, the application of properties such as softness and self-healing is extremely valuable. As a gel electrolyte, it greatly promotes the development of safety and environmental protection in the traditional battery industry, and can better promote the progress of the new energy industry represented by trams.

Cellulose-based conductive hydrogel is a flexible and conductive material that simultaneously exhibits excellent mechanical properties and versatility, making it an ideal candidate for use in flexible electronics and bioelectronics. The applications of cellulose-based conductive hydrogel mainly include the gel as all the components to form a cellulose-based conductive hydrogel electronic system and cellulose-based conductive hydrogel as a flexible electrode applied to the connection of rigid devices in these two categories. The potential applications of cellulose-based conductive hydrogels in a variety of fields are illustrated in Figure 5. This paper addresses the development and application of cellulose-based conductive hydrogels in four key areas: wearable sensors, smart biomedicine, supercapacitors, and gel electrolytes in conventional batteries.

### 4.1. Wearable Sensors

The advent of flexible wearable devices that are soft and form-fitting has led to the development of innovative solutions for real-time monitoring of human health. These devices have the potential to overcome the incompatibility between electronics and human biology, which has previously hindered the development of effective health-monitoring solutions [110]. The miniaturization, low energy consumption, sensitivity, and multi-functional intelligence of wearable sensors have been the subject of continuous improvement and perfection. Furthermore, the ability to combine machine learning and artificial intelligence has also been well applied, which will facilitate the non-invasive dynamic monitoring of various types of electrical signals (motion, temperature, sweat, etc.). This will also serve to advance the internet healthcare model. Cellulose-based conductive hydrogel exhibits favorable biocompatibility and degradability, along with readily adjustable mechanical properties and commendable electrical conductivity. This makes it an exemplary candidate for incorporation into wearable devices, and it has demonstrated significant advancements in this field [111,112,113].

The unique properties of cellulose-based hydrogels, such as their high water content and excellent biocompatibility, make them ideal candidates for fabricating breathable on-skin electronic tattoos. For example, by modifying the surface of cellulose-based hydrogels with specific functional groups, it is possible to enhance their adhesion to the skin while maintaining breathability. This not only improves the comfort of wearing on-skin electronic tattoos but also ensures long-term stable performance.

Conventional hydrogels, as soft and weak materials, frequently present difficulties in application and promotion due to their inability to meet the requisite mechanical strength standards. However, cellulose is frequently employed as a hydrogel-reinforcing agent, and the numerous hydroxyl and carboxyl groups on the surface can be utilized to form hydrogen bonds or chemically modified to construct conductive hydrogels with high mechanical properties, which can then be used for the preparation of wearable sensors. As Song et al. prepared multi-CNCs (CNC-g-AA-g-CA) by hydrolyzing MCC with hydrochloric acid/citric acid, aniline and hydrochloric acid were subsequently added to the multi-CNCs to create multi-CNC–PANI. Finally, the multi-CNC–PANI hydrogel was prepared by adding a PVA solution and borax. Multi-CNC–PANI, PVA/PANI, and borax were added to the multi-CNCs. The combination of PANI, PVA/borax dynamic hydrogen bonding interactions, and dynamic borate bonding resulted in the hydrogel exhibiting enhanced mechanical properties. As an illustrative example, the tensile amplitude of 5 wt% multi-CNC–PANI/PVA can exceed 1500% in a mechanical test, with corresponding values for tensile strength, Young’s modulus, and tensile strain of 171.52 kPa, 2086.91 kPa, and 1085%, respectively. The self-healing rate reaches 99.56% within 120 s, with the electrical conductivity remaining stable after the healing process, demonstrating exceptional self-healing performance. Additionally, the favorable adhesion, biocompatibility, and sensitivity, coupled with the capacity for stable strain signal monitoring, render it an optimal selection for the fabrication of wearable strain sensors for the observation of bodily movement [114].

As demonstrated above, the incorporation of conductive hydrogels with excellent self-healing capabilities will markedly enhance the durability and signal stability of the material. The self-healing ability of hydrogel enables the recovery of mechanical damage sustained during daily use, thereby prolonging the service life. This is a significant distinction from traditional rigid sensors. Sun et al. prepared cellulose-based ionic conductors (ICs) using a green one-pot method. This involved the addition of ChCl and AA to pretreated kraft pulp paper, which was then rapidly hydrolyzed to obtain CMFs. Subsequently, in situ photopolymerization was carried out with direct light, resulting in the formation of multifunctional PDES/CMF ICs. Multifunctional PDES/CMF ICs were successfully obtained. In the tests, the PDES/CMF ICs exhibited excellent mechanical properties, including high elongation (3210 ± 302%) and toughness (13.17 ± 2.32 MJ·m^−3^). Nevertheless, the most notable attribute remains the exceptional fatigue resistance and self-recovery capabilities, which are also crucial prerequisites for signal stability and a guaranteed lifetime. In cyclic stress–strain experiments, PDES/CMF ICs demonstrated remarkable resilience, retaining 57% of the original stress after 100 loading–unloading cycles. Furthermore, the PDES/CMF ICs exhibited an exceptional capacity for recovery, almost returning to their original length after 30 min of rest following 100 cycles. This highlights their excellent dissipated energy-recovery and stress-recovery capabilities. The cut PDES/CMF ICs demonstrate a continued healing process over time, with the resistance value returning to its original value and the tensile stress recovering to approximately 100%. In addition to exhibiting good transparency, ensuring the transmission of optical signals, and high strain sensitivity, these materials can be applied to wearable sensors for the detection of human motion and physiological signals. These straightforward and environmentally friendly techniques will facilitate the creation of resilient, self-healing, transparent, and long-lasting ICs, while also offering a novel perspective on sustainable synthesis processes for cellulose-based flexible electronics [115].

In addition to the self-healing property, the reliable power supply also severely limits the application of cellulose-based conductive hydrogels in the field of flexible electronics. The self-supply of conductive hydrogel power represents an excellent solution to this problem. As Qin et al. oxidized CNFs with TEMPO to obtain TOCNF, and then proceeded to dynamically cross-link and polymerize PANI and TOCNF with PVA/borax in situ, they were able to obtain a self-powered conductive hydrogel with high stretchability and self-healing ability, which they designated the TOCNF/PANI–PVAB hydrogel (CPPH). The CPPH hydrogel has a stretchability of up to 1530%, and the excellent self-healing ability gives it a good ability to supply electricity. The CPPH hydrogel exhibits a tensile strain of up to 1530%, coupled with an exceptional self-healing capacity that enables the restoration of its electrical conductivity within 10 s, with a self-healing efficiency exceeding 95%. Its intrinsic conductivity reaches 0.6 S·m^−1^. Polydimethylsiloxane (PDMS) is employed as the negative friction layer and encapsulation material to assemble with the CPPH hydrogel to form a sweat sensor. The high negative triboelectricity of PDMS provides a larger electrical output for the CPPH sweat sensor, enabling more sensitive reflection of changes in target ion concentration. A real-time quantitative analysis of Na^+^, K^+^, and Ca^2+^ in sweat was performed based on the triboelectric effect, with sensitivities of 0.039, 0.082, and 0.069 mmol^−1^, respectively. The results were stably transmitted wirelessly to the user interface. The use of CPPH hydrogels in the assembly of flexible sweat sensors represents a promising avenue for the development of self-powered sweat health-monitoring solutions [116]. These sensors offer a unique combination of high flexibility, stability, analytical sensitivity, and selectivity, positioning them as a competitive option in the future health-monitoring landscape.

In order to more accurately represent the reactive properties of cellulose, a number of the functionalization strategies of cellulose previously mentioned were adopted for the purpose of advancing the development of wearable sensors. For instance, Shi et al. oxidatively modified cellulose to produce dialdehyde cellulose (DAC), and then utilized the amino groups in gelatin and DAC to undergo a Schiff base reaction, which ultimately resulted in a PAM/DAC-2Gel hydrogel. The presence of a reduced number of hydroxyl groups in DAC compared to ordinary cellulose has been demonstrated to result in a decrease in the content of free water, and consequently, an enhancement in the hydrogel’s mechanical strength and adhesive properties. Concurrently, the double cross-linking network structure formed by this hydrogel contributes to the increase in bound water content. This modulation of the change in free and bound water content in the hydrogel has been shown to result in interfacial adhesion and conductive sensing hydrogel materials that are resistant to humid environments. The addition of Na+ has been shown to enhance the sensitivity of the PAM/DAC-2Gel hydrogels, ensuring the stability and reproducibility of their sensing performance, resulting in a fast response time and consistent sensing performance. The superior adhesion and biocompatibility exhibited by the PAM/DAC-2Gel hydrogels suggests that they have great potential for application in the fields of flexible electronic sensors and electronic skin, which are expected to be used for future human health monitoring. This strategy of endowing cellulose with functionalization has been shown to reach the ability of double cross-linking and regulating free and bound water. This provides a reference for the application of modified cellulose in the field of conductive hydrogels [117]. Furthermore, graft modification represents the most prevalent functionalization strategy for cellulose, a fact that renders it of significant research value. For instance, Shi et al. successfully grafted poly(lysine) (PLL) onto the surface of CNCs through electrostatic interaction to create CNCs@PLL, and then added ZP (a cyclic subamino acid), PAA, and Fe^3+^ to form PCZFe hydrogel. PAA exhibits various noncovalent interactions with CNCs@PLL, ZP, and Fe^3+^, thereby endowing PCZFe hydrogel with excellent mechanical properties and self-healing properties. PAA has been shown to interact with CNCs@PLL, ZP and Fe^3+^ via a variety of non-covalent mechanisms. This interaction contributes to the exceptional mechanical and self-healing properties of the PCZFe hydrogel. Furthermore, the PCZFe hydrogel sensor demonstrates excellent potential for use in low-temperature environments, exhibiting mechanical strength (2.9 MPa), self-healing (87.3%), and electrical (2.0 S/m) properties at −20 °C [118]. The significance of graft-modified cellulose in wearable sensors and solid-state batteries, biomedicine, and other fields is also highlighted. It is anticipated that scholars will develop further cellulose functionalization strategies to further promote the utilization of cellulose in various industries [119,120,121].

### 4.2. Intelligent Biomedicine

Cellulose-based conductive hydrogel, a type of biomass-based hydrogel, is non-toxic and non-irritating. Its excellent biocompatibility and conductivity facilitate a connection between human biology and electronics, offering a novel solution for monitoring physiological information and personalized health. In addition to wearable devices, the diverse applications and performance of cellulose-based conductive hydrogel will provide the medical business with new medical strategies in the AI era. These include smart wound dressings, e-skin, bionic materials, implantable soft robots, and the monitoring of bioelectrical signals. The hydrogel also plays a role in supporting anti-bacterial, cell proliferation, and other effects. The advent of flexible electronics has led to the rapid development and innovation of a plethora of new intelligent biomedical technologies, which will undoubtedly serve to advance the field of medicine and promote human health through the integration of the Internet of Things and artificial intelligence [109,122,123].

Wound healing represents the most common and significant medical necessity. The process of wound healing is dependent on the coordinated interaction of four distinct phases: hemostasis, inflammation, proliferation, and remodeling [124]. While minor skin wounds may be capable of self-healing, severe wounds, including those caused by traffic accidents, surgical incisions, and other serious injuries, can result in significant damage that is challenging to heal [125]. In such cases, the presence of bacteria or infection can impede the natural healing process, potentially leading to disability or even death. One may consider traditional wound dressings, such as Band-Aids, bandages, and medical gauze, which are primarily composed of fabric and adhesive. Despite the capacity of traditional fabric-based wound dressings to absorb tissue exudate from the wound, the occurrence of adhesion and infection at the wound site remains a common phenomenon. The difficulty in judging drug release, the lack of reusability, and the risk of infection when replacing multiple times are all significant challenges that are difficult to overcome with traditional dressings. Hydrogel wound dressings possess the capacity to provide a moist environment conducive to healing, akin to that of native tissue, while simultaneously absorbing exudate and facilitating optimal air–liquid exchange. Their prolonged service life, ease of re-administration of drugs, and ability to monitor drug release and the real-time health status of the wound through electrical signals make them an ideal choice for new wound dressings [126]. Furthermore, the development of conductive hydrogel allows for the provision of intelligent services for wound healing through the utilization of electrical signals. Cellulose-based conductive hydrogel, exemplified by conductive hydrogel, exhibits favorable biocompatibility and an absence of allergic reactions. Furthermore, the modified cellulose displays promising antimicrobial properties and the capacity to stimulate cell proliferation, in conjunction with the conductivity of the electrical signals, rendering it an optimal selection for the fabrication of intelligent wound dressings. As Li et al. initially modified cellulose nanocrystals (CNCs) using tris(2-carboxyethyl) phosphine oxide (TA) to obtain TA@CNC, they then added this to a mixed solution of water and glycerol. Following the introduction of an appropriate quantity of FeCl_3_·6H_2_O into the combined solution, robust adhesive and self-healable PTCGFe hydrogels with self-powered characteristics were generated through the final polymerization process, which involved the addition of AA, MBAA, and APS. The resulting PTCGFe hydrogel demonstrated good mechanical properties, with a tensile strength of 63 kPa and a 527% strain. The hydrogel exhibits an excellent self-healing ability of up to 91.6% due to the abundance of hydrogen bonding and reversible metal–ligand bonding with Fe^3+^. The PTCGFe hydrogel is self-powered, as demonstrated by the stable supply of power to the LEDs in the experiments, which was achieved through the use of a fruit battery inspired by the PTCGFe hydrogel. The natural nature of TA has enabled the hydrogel to demonstrate favorable results in antimicrobial, cytotoxicity, and in vivo antimicrobial healing experiments, thereby meeting the requirements for wound dressing applications. Concurrently, the sensitive and stable real-time monitoring of movement and the utilization of electrical signals to characterize drug release can be repeated, and the electrical signals remain stable and almost unchanged after multiple administrations. The intelligent dressing constructed with PTCGFe hydrogel, which exhibits good adhesion, in vivo antimicrobial properties, and in vivo wound healing ability, not only improves the efficiency of wound healing but also strengthens health monitoring [127]. Furthermore, it promotes the combination of wound healing and the internet, with the wireless and stable transmission of signals. This has broad application in the field of intelligent medical treatment in the future.

Composite cellulose-based conductive hydrogels have a variety of potential applications, including use in in vitro wound dressings, in vivo injections, and tissue engineering. To illustrate, Patel et al. synthesized CMCS after extracting s-NCs and CNCs from pine powder. They then created a CMCS/s-NC composite conductive hydrogel and applied it to porous scaffolds. The conductivity of the CMCS/s-NC composite conductive hydrogel was increased to 2.892 × 10^−3^ S/cm, which has the potential to significantly stimulate the acquisition of skin cells and accelerate wound healing following implantation into the skin. The hydrogel scaffold supported the growth of three types of cells, namely human dermal fibroblasts (HDFs), human keratinocytes (HaCaTs), and human umbilical vein endothelial cells (HUVECs), and facilitated healthier cell culture. Furthermore, CMCS/s-NC composite hydrogel scaffolds enhanced gene expression and cellular activity, with the scaffold-treated HUVEC-related gene markers exhibiting heightened expression. Notably, the expression of vascular endothelial growth factor (VEGF), VE–cadherin (VE–CAD), and CD31 was elevated in processes related to the vasculogenesis, maintenance, and organization of the vascular structure. The generation, maintenance, and organization of the vascular structure play an important role in this process. This hydrogel scaffold is highly efficient and promotes angiogenesis. Furthermore, the hydrogel stimulates granulation tissue and facilitates rapid wound healing by increasing tissue thickness. The hydrogel’s favorable antimicrobial and biocompatibility properties, coupled with its potential for wireless transmission of electrical signals via its good electrical conductivity, make it a promising candidate for monitoring tissue repair and gene expression in vivo [128].

Furthermore, cellulose-based conductive hydrogels have the potential to be utilized in more intelligent medical fields. Conductive hydrogels can serve as a conduit for an external electric field, facilitating the use of electrical stimulation to achieve a desired medical outcome. For instance, Yan et al. employed MCC to extract CNF from a PGCNSH conductive hydrogel. This approach enables the in vivo delivery of electrical stimulation, which can enhance the repair of diabetic wound trauma by coupling with external electrical stimulation. This represents a promising avenue for electrotherapy, as it can promote cell proliferation and differentiation. Conductive hydrogel can also be employed as a cutting-edge alternative in the regeneration of nerves and other medical fields [129]. Xu et al. utilized cellulose with favorable biocompatibility and mechanical properties of PANI/cellulose conductive hydrogel, demonstrating its efficacy in guiding the regeneration of the sciatic nerve of a rat model without additional treatment. This has significant potential in the biomedical materials field [130].

The conductive function of cellulose-based conductive hydrogel enables the provision of a conductive microenvironment, which facilitates electrical communication and thus promotes tissue regeneration. Furthermore, it serves as a bridge to enhance communication and electrical coupling between normal and damaged tissues. The combination of this with wireless transmission of signals allows for intelligent monitoring, which can be used to play an intelligent medical treatment role in human bone repair, nerve tissue regeneration, and artificial organs, among other applications. This has the potential to empower various medical fields in the future.

### 4.3. Flexible Supercapacitors

The accelerated evolution and integration of flexible electronic devices, coupled with the necessity for low-energy consumption and a reliable energy supply, has rendered this an increasingly crucial and pressing concern. In addition to self-powered energy supply, flexible supercapacitors offer the potential for enhanced stability and prolonged output power. The principal components of supercapacitors are electrodes, a diaphragm, a fluid collector, and an electrolyte, which are in contrast to the constituents of batteries. The charge transfer rate of supercapacitors is faster, and they are capable of more frequent charge/discharge cycles. In comparison to traditional supercapacitors, flexible supercapacitors, as energy storage elements, are capable of meeting the demands of flexible electronic devices with regard to movement and bending. In addition, flexible supercapacitors possess the following advantages: they are lightweight, have a long life cycle, exhibit high power density, are self-healing, are inexpensive and environmentally friendly, and are capable of integrating rigid, high-precision, micro-small devices while simultaneously providing energy [131,132,133].

The potential for recyclable and biodegradable supercapacitors to be utilized in a variety of contexts within the field of green energy storage is significant. Cellulose is a non-toxic, biodegradable, and renewable biopolymer material that can be readily modified into cellulose derivatives containing various functional groups. These derivatives can be used in the field of energy storage, with particular potential for composites comprising cellulose as the matrix and combined with conductive materials, which exhibit enhanced electrical properties. As Wan et al. dissolved cellulose in a BzMe_3_NOH aqueous solution and added a cross-linking agent, PEGDE, to obtain a cross-linked cellulose, the cellulose was then immersed in a CPA solution to obtain a CP hydrogel. Finally, the CP hydrogel was immersed in an aniline/PA solution to obtain a flexible, tensile-property sandwich-shaped hydrogel with high conductivity, namely a cellulose/PA/PANI hydrogel. The addition of PA and PANI resulted in an enhancement of the tensile strength of the CPP. The addition of PA and PANI resulted in an enhancement in the tensile strength (0.78 MPa) and tensile properties (elongation at break of 27.00 ± 5.12%) of CPP. The excellent mechanical properties of CPP enabled the integrated supercapacitor made from CPP to maintain good capacitance performance under deformation, particularly in terms of capacitance durability, which only reduced to 89% after 1000 bending cycles. This is indicative of good stability under mechanical loading. Furthermore, PANI particles not only effectively reduce the interfacial resistance between the electrode and electrolyte, but also the interaction between cellulose, PA, and PANI significantly enhances the electrical conductivity through the doping effect, while simultaneously dissipating the mechanical energy generated by deformation. The integrated supercapacitor exhibited a high capacitance value of 1210.7 mF·cm^−2^, a maximum energy density of 168.2 μW·h·cm^−2^, and a maximum power density of 669.1 μW·cm^−2^ at a current density of 1 mA·cm^−2^. The CPP is a versatile material that can be employed in the fabrication of supercapacitors and highly sensitive strain sensors for the monitoring of body movement. The hydrogel can be further coupled with advanced electronic devices, which will facilitate its use in self-powered devices [134].

In comparison to traditional capacitors and batteries, flexible supercapacitors with cyclic self-healing ability have the potential to be applied in a wider range of scenarios and are more competitive. For example, Lin et al. added CA to CMC solution under stirring conditions, and subsequently immersed the hydrogel into a CaCl_2_ solution to obtain a DP CMC hydrogel with enhanced toughness, self-healing, and conductive properties. The hydrogel has a dual physical cross-linking structure. The H^+^ of CA partially protonates the –COO– of CMC, forming –COOH and a multitude of carboxylate hydrogen bonds. Concurrently, Ca^2+^ is uniformly dispersed in the hydrogel, further cross-linking the –COO– group. This endows the DP CMC hydrogel with robust mechanical properties and self-healing CMC. The doping of Ca^2+^ is also a principal factor contributing to the conductivity of the hydrogel, with an ionic conductivity of 6.42 S/m. The mechanical test revealed that the maximum compressive fracture stress was 4.42 MPa, while the maximum fracture energy was 18.6 KJ·m^−2^. The self-healing performance test demonstrated that the cut hydrogel can be healed rapidly, within five seconds. The supercapacitors prepared in this study demonstrate high specific capacitance (309 F·g^−1^) and volumetric capacitance (2.60 F·cm^−3^). It is anticipated that this all-cellulose-based supercapacitor will be employed as a functional sustainable electronic material when combined with super toughness, rapid self-healing ability, and fast degradation ability. This makes it a highly competitive option in the field of green functionality [135].

The electrolyte of supercapacitors is susceptible to environmental factors, with temperature representing a significant influence on the charging and discharging performance. In order to enhance the practical applicability of cellulose-based supercapacitors, it is imperative to optimize the temperature range in the development of flexible supercapacitors. As described by Zhang et al., the sequential addition of LiCl, monomer AM, APS solution, and MBA solution to a preformed water-soluble cellulose acetate (WSCA) aqueous solution resulted in the formation of an anti-freezing and anti-dehydration conductive hydrogel, namely the PAM/LiCl/WSCA hydrogel, which exhibited robust mechanical properties. The rGO hydrothermal gel can be compressed into two GO electrodes, and the PAM/LiCl/WSCA hydrogel can then be employed as the electrolyte to create a flexible supercapacitor with a sandwich-like structure. The rGO hydrothermal gel exhibits excellent mechanical properties, with a tensile strength of 341 kPa and a specific energy density of 1.2 MJ/m^3^. One of the material’s primary advantages is its wide operating temperature range. The wide operating temperature range is one of the material’s main advantages. The LED can be lit in the test range of −80 °C to 80 °C, and it exhibits high conductivity (16.7 S/m at 25 °C) and low conductivity (2.4 S/m at −40 °C) at different temperatures. Notably, it maintains a certain degree of toughness and elasticity at −80 °C (no phase transition occurs), which is indicative of its antifreeze performance. The assembled flexible supercapacitor also demonstrated excellent capacitance stability in 500 folding cycles and 10,000 charge/discharge tests, retaining 64.64% of its capacitance at −40 degrees Celsius. This straightforward approach to creating freeze-resistant conductive hydrogels with superior mechanical resilience is highly beneficial in expanding the operational temperature range [136]. It also offers a novel concept for the development of eco-friendly flexible energy storage devices that can withstand harsh conditions.

### 4.4. Application of Gel Electrolytes in Conventional Batteries

Cellulose-based conductive hydrogel has a significant impact on the development of emerging intelligent technologies such as flexible electronics. Additionally, it is a crucial enabler in the advancement of traditional electrical industry sectors. The battery industry provides an illustrative example. Lithium batteries possess exemplary properties, including high energy density, high operating voltage, and rapid charging and discharging. They are the primary energy supply devices in aerospace and electric vehicles, among other applications. However, the liquid electrolyte is susceptible to leakage and flammability, and Li^+^ is prone to accumulation in the vicinity of the diaphragm holes, which results in the uneven deposition of Li^+^ on the surface of the anode, thereby facilitating the growth of lithium dendrites. The formation of lithium dendrites represents a significant challenge to the advancement of lithium batteries. In addition to accelerating the consumption of electrolytes and compromising the integrity of the electrolyte layer on the surface of the anode, lithium dendrites may also puncture the diaphragm, leading to a short-circuit of the battery and, consequently, a series of major safety incidents [137]. Consequently, the utilization of gel electrolyte (GPE) in lieu of liquid electrolyte serves to mitigate the risk of liquid leakage and maintain a stable electrochemical window. Furthermore, it represents an optimal strategy for addressing the growth of lithium dendrites [138]. Zhao et al. obtained a cellulose/PEG hydrogel and employed it as a novel polymer gel electrolyte. This was achieved by first dissolving cellulose in a NaOH/urea solution, followed by the addition of polyethylene glycol (PEG). Finally, 1,2-ethanediamine hydrochloride (ECH) was used as a cross-linking agent. The mechanical and ionic transport properties of this GPE can be regulated by modifying the PEG content. The results of the experimental investigation indicate that the tensile strength could reach up to 211.06 MPa, while the lithium-ion conductivity could reach up to 3.31 × 10^−3^ S/cm. The highest lithium-ion conductivity was observed in the 5% PEG content sample, with an iron mobility number of 0.63. When assembled as a lithium/GEG/NCM523 battery, the initial discharge capacity at 0.2C was found to be 159.3 mAh·g^−1^, with a Coulombic efficiency of 85.52%. The excellent performance of cellulose/PEG gel-based GPE, coupled with its straightforward and environmentally benign synthesis process, renders it a highly competitive alternative to conventional electrolytes [139].

In addition to lithium-ion batteries, zinc-ion batteries (ZIBs) are a hot topic in battery research, but the use of zinc metal as an anode is often associated with dendrite growth and corrosion passivation. Therefore, the development of cellulose conductive gel-based GPEs is also of great research value for the further development of ZIBs [140,141]. For example, Xu et al. dispersed commercial cellulose nanofiber gel in deionized water and added a certain amount of CMC solution, and then the dispersion was successively immersed in NaOH solution and ZnSO4 solution after vacuum filtration to obtain a highly flexible and hydrophilic cellulose–CMC hydrogel electrolyte. The CMC contained in this GPE forms strong interactions with water through –COOH, which can play a role in increasing the bound water content and limiting the free water content. An adequate bound water content helps to ensure the conductivity of Zn^2+^, and the reduction in the free water content also avoids the occurrence of a number of side reactions, such as passivation. The amphiphilic cellulose also provides specific channels for ion transfer, which largely avoids disordered accumulation of Zn^2+^. NaOH has a mechanical strengthening effect on the cellulose–CMC hydrogel electrolyte, the tensile strength is increased to more than 70 MPa, and the combination of strong mechanical strength and water-binding ability ensures cycle stability and high electrical conductivity (26 mS·cm^−1^). With the cellulose–CMC hydrogel electrolyte, the Zn anode maintains a current density of up to 80 mA·cm^−2^ for more than 3500 cycles, and a cumulative capacity of 17.6 A·h·cm^−2^ can be achieved at a current density of 40 mA·cm^−2^. The electrolytes exhibited excellent electrochemical and cycling performance when used to assemble Zn||MnO_2_ batteries. The cellulose–CMC hydrogel acts as both a quasi-solid electrolyte and a diaphragm, and the simple and environmentally friendly fabrication process, as well as the good applicability of the electrolytes, make them suitable for use in Zn||MnO_2_ batteries. With the cellulose–CMC hydrogel electrolyte, the Zn anode maintains a current density of up to 80 mA·cm^−2^ under more than 3500 cycles, and a cumulative capacity of 17.6 A·h·cm^−2^ can be achieved at a current density of 40 mA·cm^−2^. The electrolytes exhibited excellent electrochemical and cycling capabilities when used to assemble Zn||MnO_2_ batteries. The cellulose–CMC hydrogel acts as a quasi-solid electrolyte as well as a diaphragm, and the simple and environmentally friendly manufacturing process as well as the good application results on Zn||MnO_2_ batteries demonstrate its great potential for application in ZIBs [142].

## 5. Summary and Outlook

Cellulose is one of the most prevalent natural macromolecules, exhibiting a diverse range of raw materials, varieties and low production costs. It has favorable biocompatibility, degradability, and mechanical properties, and the numerous active sites on the surface can also be modified to yield functional groups and a range of functional cellulose derivatives. Furthermore, cellulose is playing a variety of roles in the field of hydrogels, with the objective of meeting the specific application needs of different areas. The addition of conductive properties to cellulose-based hydrogels has the potential to make them an important component in flexible electronic materials, which could be used in a number of fields, including smart medical care, human body sensing, and green energy storage. Specifically, these hydrogels have the potential to be used as comfortable, close-to-the-human-body, real-time sensors; for the visualization of information and intelligent biomedical applications; for flexible, self-healing supercapacitors; and to assist in the development of the traditional battery industry. These findings illustrate the considerable potential of cellulose-based conductive hydrogels for future applications.

However, there are numerous challenges that must be overcome to integrate cellulose-based conductive hydrogels more effectively with multiple functions, achieve enhanced multifunctionality and intelligence, establish a close link between the human body and artificial intelligence, and facilitate the continued advancement of artificial intelligence in the new era. Furthermore, the achievement of simple, green, and intelligent large-scale industrial generation of cellulose-based hydrogels is also challenging and necessitates the collective efforts of numerous individuals to facilitate the early commercialization of these products.

If the practical applications of cellulose-based conductive hydrogels in flexible electronics, biomedicine, and energy storage are to be better met in the future, there are many actions that can be taken to achieve this goal. The modification of cellulose or the construction of a new hydrogel network structure can balance the relationship between mechanical properties and functionality; the use of conductive fillers, ionic solutions, chemical reactions, etc. can promote the development new conductive strategies and further the long-term stability and sensitivity of conductive materials; and strengthening the combination of emerging production processes such as 3D printing and conductive hydrogel can further satisfy the synergism between production and performance development.

## Data Availability

Not applicable.
